# 3 × 40 Gbit/s All-Optical Logic Operation Based on Low-Loss Triple-Mode Silicon Waveguide

**DOI:** 10.3390/mi13010090

**Published:** 2022-01-07

**Authors:** Yuhang Hu, Zihao Yang, Nuo Chen, Hanwen Hu, Bowen Zhang, Haofan Yang, Xinda Lu, Xinliang Zhang, Jing Xu

**Affiliations:** School of Optical and Electronic Information, Huazhong University of Science and Technology, Wuhan 430074, China; yuhanghu@hust.edu.cn (Y.H.); m202172642@hust.edu.cn (Z.Y.); nuochen@hust.edu.cn (N.C.); hwhu@hust.edu.cn (H.H.); M202072632@hust.edu.cn (B.Z.); haofan_yang@hust.edu.cn (H.Y.); xindalu@hust.edu.cn (X.L.); xlzhang@mail.hust.edu.cn (X.Z.)

**Keywords:** all-optical signal processing, spatial mode multiplexing, optical logic device, four-wave mixing

## Abstract

Information capacity of single-mode fiber communication systems face fundamental limitations imposed by optical nonlinearities. Spatial division multiplexing (SDM) offers a new dimension for upgrading fiber communication systems. Many enabling integrated devices, such as mode multiplexers and multimode bending with low crosstalk, have been developed. On the other hand, all-optical signal processing (AOSP) can avoid optical to electrical to optical (O–E–O) conversion, which may potentially allow for a low cost and green operation for large-scale signal processing applications. In this paper, we show that the system performance of AOSP can be pushed further by benefiting from the existing technologies developed in spatial mode multiplexing (SDM). By identifying key technologies to balance the impacts from mode-dependent loss, crosstalk and nonlinearities, three-channel 40 Gbit/s optical logic operations are demonstrated using the first three spatial modes in a single multimode waveguide. The fabricated device has a broadband four-wave mixing operation bandwidth (>20 nm) as well as high conversion efficiency (>−20 dB) for all spatial modes, showing the potential for a large-scale signal processing capacity with the combination of wavelength division multiplexing (WDM) and SDM in the future.

## 1. Introduction

All-optical signal processing (AOSP), which ensures that optical signals are directly processed by another beam of light without passing into the electrical domain, is a long-standing goal for developing energy-efficient optical interconnection and optical communication systems [1,2]. The need for advanced AOSP is urgent due to the rapid growth of bandwidth-hungry applications and the bottleneck of signal delivery, and processing becomes apparent in data center and optical routing nodes. AOSP technologies, including, but not limited to, wavelength conversion, amplitude, signal reshaping, all-optical logic and phase regeneration [3,4], have shown massive promising prospects. They benefit from the ultrafast response time of optical nonlinear effects, e.g., four-wave mixing (FWM), resulting in the data signal being processed on-the-fly. Meanwhile, AOSP breaks the limitation of signal modulation speed in optic-to-electric (O/E) conversion or vice versa, overcoming the (de)modulation complexity in electrical signal processing thanks to the modulation-format-transparent feature of AOSP. It is also highly integrable and scalable on various material platforms based on matured integration technology. Therefore, integrated AOSP means potentially low power consumption and complexity, which is significant to the eternal theme of development for information technology.

On the other hand, various dimensions have been exploited to promote the capacity of optical communication systems; for instance, time-division multiplexing (TDM) [5] and wavelength-division multiplexing (WDM) [6]. Applying advanced modulation formats is also a meaningful way to increase the capacity of the optic fiber communication systems. In recent years, many works have demonstrated that the spatial dimension can be used as a new dimension to increase the transmission capacity of a single fiber or on-chip single waveguide [7]. Spatial-division multiplexing (SDM) or mode division multiplexing (MDM), which refers to multicore/multimode optical fibers or multimode waveguides, has recently attracted intensive attentions. Different signals carried on the different orthogonal eigenmodes can be transmitted or processed in one single fiber/waveguide in parallel, yielding a significant growth of communication capacity [8]. Importantly, silicon photonics, which take advantage of compact and low loss, can leverage the existing infrastructure that has been developed for the semiconductor industry. Mode multiplexer is particularly important in on-chip optical interconnection, which has been realized based on various structures, such as multimode interferometers [9], asymmetric Y-junctions [10], adiabatic couplers [11] and asymmetric directional controller (ADC) [12]. By harnessing the high-order mode Kerr nonlinear effect, matured MDM technologies can be combined with AOSP to expand signal processing capacity. It is known that the intra-mode phase-matching conditions are easy to meet, but the phase-matching conditions between different modes are difficult to satisfy. Therefore, when the pump and signal light are in the same spatial mode, strong FWM will occur [13] while nonlinear crosstalk between different modes can be kept small. Combined with advanced modulation formats, the all-optical wavelength conversion of 102.6 Gbit/s orthogonal frequency division multiplexing- quadrature phase shift keying (OFDM-QPSK) signals has been verified [14]. However, the conversion efficiency of FWM is only −33 dB, and the conversion bandwidth is only 0.6 nm. In this work, a multimode silicon waveguide that supports three transverse-electric (TE) spatial modes is designed and fabricated, achieving an FWM conversion efficiency of −20 dB and conversion bandwidth of >20 nm for all three modes. By using a dual pump FWM configuration and encoding data A and B onto two pump lights, the generated idlers carry the AND logic result (A&B). This is because idler light can only be generated when signal light A and pump light B have power simultaneously.

Table 1 summarizes the all-optical logic devices reported so far based on multimode photonic integration circuits. Our work clearly shows the utilization of the highest number of modes and achieves a 3 × 40 Gb/s data capacity, offering the potential for updating the all-optical signal processing capacity with MDM technology.

## 2. Design of Low-Loss Triple-Mode Nonlinear Waveguide

**Figure 1:**
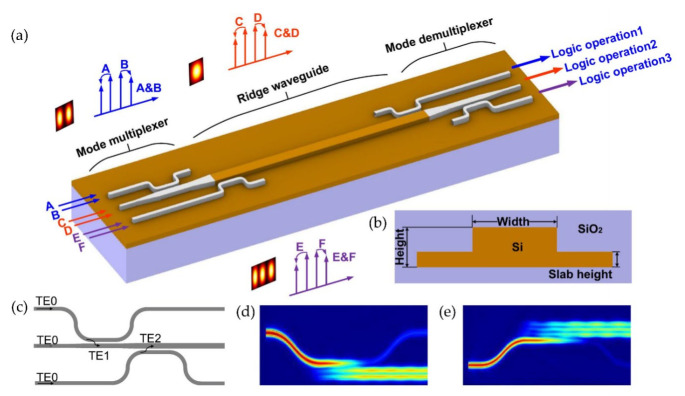
(**a**) Schematic diagram of on-chip three-channel all-optical logic operations. Top silica cladding is not shown for clarity. (**b**) Sketch of ridge waveguide on SOI platform. The height of the ridge waveguide is 220 nm. (**c**) A three-mode multiplexer based on asymmetric directional controllers (ADCs). (**d**,**e**) Light propagation in the coupling regions of the multiplexer when the launched TE0 mode of the access waveguide couples to the TE1 (**d**) and TE2 (**e**) mode in the bus waveguide.

The principle of a chip-integrated multimode optical logic operation is illustrated in Figure 1a. A ridge multimode waveguide was designed on an Silicon-on-Insulator (SOI) platform. A mode multiplexer based on ADCs was used to couple three input channels to the TE0, TE1 and TE2 mode of the multimode waveguide. Idlers that carry the AND logic of three different channels were directed to other ports according to their spatial modes after strong intra-modal FWM in the multimode waveguide. In the context of applying Kerr nonlinear effects in the integrated waveguide, loss, dispersion and Kerr nonlinearity were at the heart of the waveguide design. The overall goals for an ideal nonlinear waveguide for AOSP were a small mode field area, low transmission loss, near-zero group velocity dispersion (GVD) and high nonlinear efficiency. In this section, we introduce the guideline for the multimode nonlinear waveguide design, disclosing the trade-off between geometric parameters.

For low-loss considerations, it has been shown that the ridge waveguide (Figure 1b) has a minor linear loss compared to the strip waveguide, benefiting from the smaller overlap between the mode field and the waveguide sidewall [17]. Meanwhile, the ridge waveguide is compatible with the p-i-n junction, which can be employed for suppressing the free carrier absorption (FCA) in the silicon waveguide. The transmission losses decrease as the slab height increases. However, the mode volume increases at the same time due to lower light confinement, which in return reduces nonlinearity. On the other hand, waveguides with highly integrated features use bending to meet the requirement of a small footprint [18]. However, scattering loss and mode crosstalk caused by bending in waveguides are fundamental problems on integrated optical platforms. Euler bending is adopted in this work to solve such issues, as it has been shown to have a great advantage at reducing bending loss as well as mode crosstalk while fulfilling small footprint requirements [19].

Dispersion plays a central role in the phase-matching condition of the FWM process. FWM efficiencies are affected by the propagation constant mismatch Δβ, which is defined as $\Delta \beta =\beta \left(\omega_{s}\right)+\beta \left(\omega_{i}\right)-2\beta \left(\omega_{p}\right)$, where $\omega_{s}$, $\omega_{i}$, $\omega_{p}$ are the angular frequencies of signal light, idler light and pump light, respectively. By using the Taylor expansion, $\Delta \beta =\beta_{2}\left(\omega_{p}\right){\left(\omega_{s}-\omega_{p}\right)}^{2}$, where $\beta_{2}\left(\omega_{p}\right)$ is the second-order dispersion of the pump light. Therefore, to ensure large conversion efficiency and large conversion bandwidth, $\beta_{2}$ needs to be near zero.

**Figure 2:**
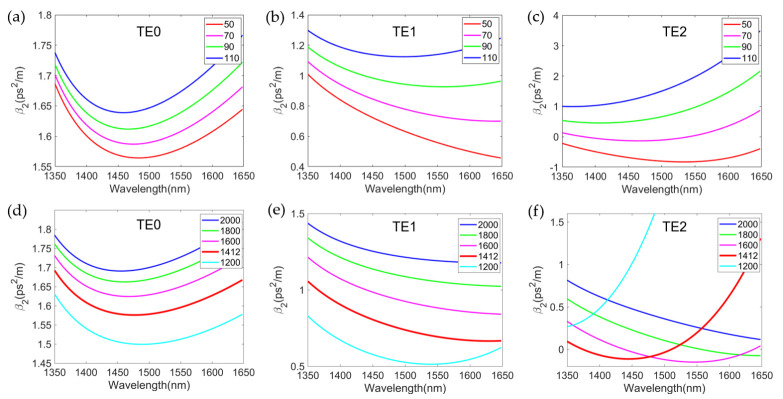
(**a**–**c**) Second-order dispersion of a silicon ridge waveguide with ridge width *w* of 1450 nm and different slab heights *h* for TE0 (**a**), TE1(**b**) and TE2(**c**) modes; (**d**–**f**) Second-order dispersion of a silicon ridge waveguide with slab height *h* of 70 nm and different ridge widths *w* for TE0 (**d**), TE1 (**e**) and TE2 (**f**) modes.

The performaces of the devices can be optimized through the control of waveguide dispersion by varying the cross-section geometry. Figure 2a–c show the relationship between the dispersion of the three modes TE0, TE1 and TE2 for different heights of the slab when the width of the ridge is chosen as 1450 nm. It can be seen that at the wavelength of 1550 nm, the second-order dispersion of the three modes decreases with the decrease of the slab height. However, a decrease of the slab height will increase the linear loss of waveguide transmission. Figure 2d–f show the relationship between the dispersion of the three modes TE0, TE1 and TE2 for different widths of the ridge when the slab height is chosen as 70 nm.

**Figure 3:**
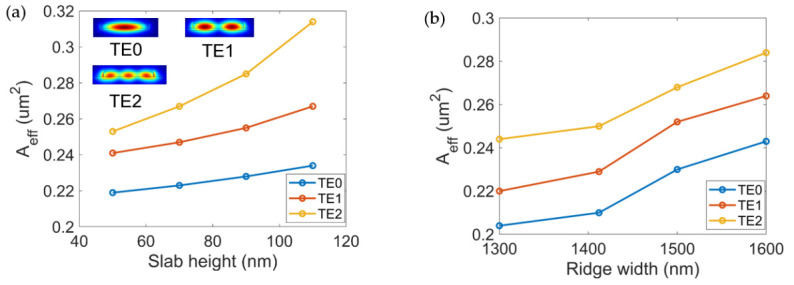
(**a**) Effective mode field area of the multimode silicon ridge waveguide as a function of slab height when the ridge width *w* is set as 1412 nm for three spatial modes. Insets: mode field distribution simulated by finite element analysis; (**b**) Effective mode field area of the multimode silicon ridge waveguide as a function of ridge width *w* for three modes when the slab heights *h* is chosen as 70 nm.

It is known that the maximum conversion efficiency of four-wave mixing is $\eta_{\max}=\gamma^{2}L_{\mathrm{eff}}^{2}P_{p}^{2}\left(0\right)e^{-\alpha L}$, where $L_{\mathrm{eff}}=\left[1-e^{-\alpha L}\right]/\alpha$ is the effective length, α is the waveguide transmission loss, γ is the nonlinear coefficient and$\;P_{p}\left(0\right)$ is the pump light power [20]. Therefore, the conversion efficiency of four-wave mixing increases with the increase of the length of the waveguide. On the other hand, the conversion bandwidth is proportional to $\sqrt{1/L}$ since $\Delta \omega =2\sqrt{\pi /L\beta_{2}}$. Therefore, compared to the increase in conversion efficiency, this decrease in conversion bandwidth is slower. The conversion efficiency of four-wave mixing is also related to the nonlinear coefficient of the material denoted as $\gamma =2\pi n_{2}/\lambda_{0}A_{\mathrm{eff}}$. Here, $n_{2}$ is the nonlinear refractive index coefficient of the material, generally valued as $6.3\times 10^{-18}\;m/w^{2}$ in the SOI, and $A_{\mathrm{eff}}$ is the effective mode field area. The smaller the effective mode area, the higher the nonlinear response. Figure 3a,b show the effective mode area of three modes as a function of the ridge width *w* and the slab height *h*. The effective mode area increases as *w* and *h* increases. Nevertheless, the increases of $A_{\mathrm{eff}}$ are very moderate.

## 3. Device Characterizations and System Performance

**Figure 4:**
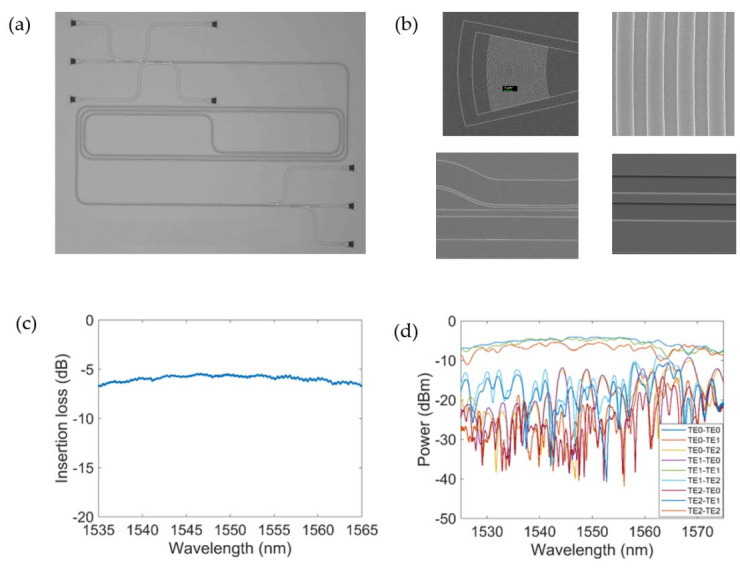
(**a**) Scanning electron microscope (SEM) image of the fabricated triple-mode all-optical signal processing (AOSP) device; (**b**) zoomed-in SEM pictures of the grating coupler, the coupling region of ADC and ridge waveguide; (**c**) measured insertion loss of the grating coupler; (**d**) measured mode crosstalk of triple-mode division multiplexing circuit.

By comprehensively considering the impact from the dispersion, loss and mode field area, we found that the waveguide width of about 1400 nm could ensure that the device has the characteristics of low loss, large conversion bandwidth and large nonlinearity. At the same time, since the phase-matching condition must be satisfied in order to excite higher order modes, the width of the multimode waveguide was found to be 1412 nm when access waveguides supporting the fundamental mode were chosen as 450 nm. To avoid further tapering length, we chose 1412 nm as the final width of the nonlinear multimode waveguide. The slab height was set as 70 nm. The gap between the access waveguide and bus waveguide was chosen as 200 nm. As a result, the lengths for the ADC coupling regions were determined as 10.5 µm and 14.5 µm for the 100% excitation of TE1 and TE2 modes according to 3D finite-difference time-domain (FDTD) simulations. Figure 1d,e show the simulated light propagation in the two designed ADCs for the TE1 and TM2 mode multiplexing, respectively. We set the mesh accuracy of the xyz axis to 20, 10 and 10 nm, respectively, and used a perfectly matched layer (PML) as the boundary condition of the area under simulation in FDTD Solution. Three different waveguide lengths were fabricated, i.e., 0.8 cm, 1.6 cm and 2.7 cm. The device was fabricated on an SOI wafer (top silicon layer: 220 nm, buried silicon layer: 2 µm), whose scanning electron microscope (SEM) image is shown in Figure 4. A 3 μm thick silica layer was deposited on the top by plasma-enhanced chemical vapor deposition (PECVD). Figure 4b shows SEM images of the grating coupler, the coupling regions for the ADCs, as well as the multimode ridge waveguide. The measured insertion loss of the grating coupler is shown in Figure 4c. Figure 4d shows the measured transmission responses at all output ports when light was launched at different input ports. At the wavelength of 1550 nm, the insertion loss of the grating coupler was about −5.5 dB, and the crosstalk between the three modes was below −10 dB.

**Figure 5:**
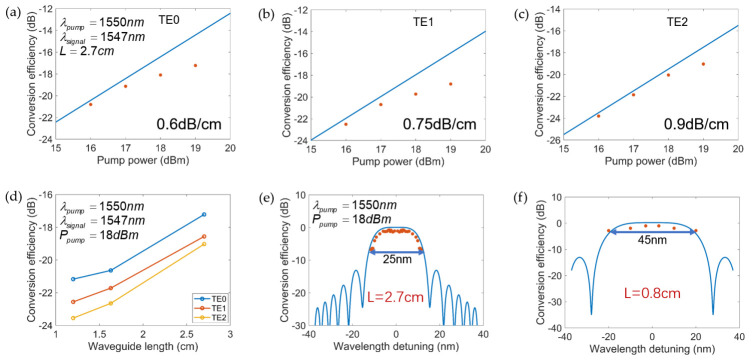
(**a**–**c**) Relationship between the four-wave mixing (FWM) conversion efficiency and the pump power for TE0, TE1 and TE2 modes when the wavelength of the signal light was 1547 nm and the wavelength of the pump light was 1550 nm in the 2.7 cm waveguide. (The blue line represents the theoretical fitting result, and the red dots represent the experimental data); (**d**) the conversion efficiency of TE0, TE1 and TE2 modes when the waveguide length was 0.8, 1.6 and 2.7 cm, respectively; (**e**,**f**) conversion bandwidth for TE0 mode at 0.8 cm and 2.7 cm waveguide length. (The blue line represents the theoretical conversion efficiency and the red dots represent the experimental data).

We conducted experiments to realize an all-optical logic operation for all the spatial modes. The pump light was set at 1550 nm and its power was amplified by an erbium-doped fiber amplifier (EDFA). The wavelength and power of signal light were 1547 nm and 10 dBm, respectively. Two polarization controllers (PCs) were introduced for pump and signal tributaries to adjust their polarization state to the same mode. A 3 dB coupler combined the tributaries of the signal and pump light, then injected them into the chip for FWM. By injecting the combined pump light and signal light to the three input ports in turn, idlers generated on TE0, TE1 and TE2 modes were demultiplexed to three output ports. Figure 5a–c show the conversion efficiency of the 2.7 cm ridge waveguide, defined as the output idler power divided by the input signal power as a function of pump power at the input of the waveguide after the vertical grating coupler. According to the analytical expression of conversion efficiency, the transmission losses of TE0, TE1 and TE2 were estimated to be 0.6 dB/cm, 0.75 dB/cm and 0.9 dB/cm, respectively, which are indeed low-loss. Due to the existence of the two photon absorption and the free carrier absorption effect, the increase of conversion efficiency was slowed down at high power levels. When the on-chip pump light power reached 18 dBm, the FWM conversion efficiency of the three modes all reached −20 dB, and the conversion efficiency of the TE0 mode could reach up to −17 dB. The impact of waveguide length on conversion efficiency is shown in Figure 5d. It is clear that as the length of the waveguide increased, the conversion efficiencies of all spatial modes increased. Figure 5e,f show that the 3-dB bandwidth of the TE0 mode reached 45 nm in the 0.8 cm long waveguide but dropped to 25 nm at a length of 2.7 cm. This confirms the trade-off between waveguide length and conversion efficiency.

**Figure 6:**
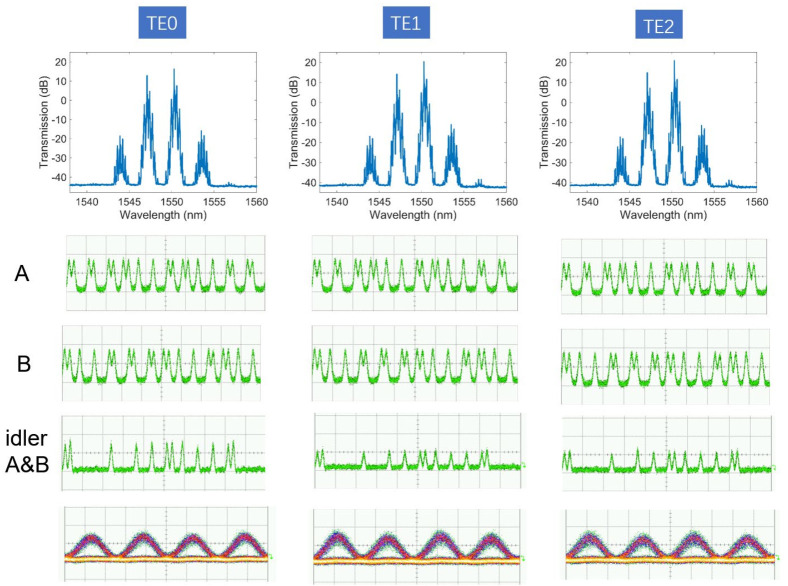
First row: Optical logic operations of the TE0, TE1 and TE2 modes; The last two rows: time domain waveform and eye diagrams of the idler generated in the three modes. (**A**,**B**) are input data while (**A**&**B**) means the logic AND operation between A and B.

In order to confirm the large signal processing capacity of our fabricated device, three channel 40 Gbit/s optical logic operations were further carried out. Two 40 Gbit/s on-off keying (OOK) optical logical data were generated by modulating two narrow linewidth continuous light at 1547.6 nm and 1550.8 nm by two cascaded MZ modulators. Two data signals, used as data A and B, were amplified by EDFA separately and then combined by a wavelength division multiplexer before sending into the device. The on-chip signal power was 20 dBm for both data streams. The FWM spectra were monitored by an optical spectrum analyzer (first row in Figure 6), and the corresponding idler light was filtered by an optical band pass filter to obtain the result of the logic operation. The FWM and logic operation of the three modes were measured separately by launching data steams once at an input port. As is shown in Figure 6, high-quality logic operation results were generated for all three spatial modes. The temporal waveforms of the AND logic of the two data signals generated in the three modes are shown in the second row of Figure 6. The last row of Figure 6 shows the fully opened eye diagrams of the idler generated in the three modes.

## 4. Conclusions

We successfully demonstrated three-channel 40 Gbit/s optical logic operations using the first three spatial modes in a single multimode waveguide. A low-loss triple-mode silicon ridge waveguide was designed and fabricated. The linear transmission loss of all three spatial modes was confirmed to be lower than 1 dB/cm. By proper dispersion engineering, the FWM conversion efficiency at −20 dB and conversion bandwidth >20 nm for all three modes were demonstrated. For system verifications, 3 × 40 Gbit/s optical AND logic operation were demonstrated using our designed triple-mode silicon waveguide with clear temporal waveform and widely open eye diagrams. To further increase the FWM conversion efficiency, p-i-n junction across the waveguide can be used. High-speed optical logic operations and all-optical signal regeneration for large capacity AOSP applications may be expected in the near future.

## Figures and Tables

**Table 1:** Performance comparison of all-optical logic operation multimode devices.

Author	Structure	Supported Mode Numbers	Logic Operation	Experiment	Signal Rate	Ref.	Year
Yunhong Ding et al.	Silicon waveguide	2	Wavelength conversion	Yes	2 × 40 Gb/s	[13]	2013
Jiamin Wang et al.	Silicon waveguide	2	AND	Yes	2 × 5 Gb/s	[15]	2017
Yonghua Wang et al.	Organic-silicon slot waveguide	2	Addition, subtraction	No	2 × 640 Gb/s	[16]	2020
Our work	Silicon ridge waveguide	FWM	AND	Yes	3 × 40 Gb/s		

## Data Availability

The data presented in this study are available from the corresponding author upon reasonable request.
